# The Late Sir Henry Burdett

**Published:** 1920-05-29

**Authors:** 


### THE LATE SIR HENRY BURDETT.

To the expressions of sympathy from public bodies already published the following should be added: From the Council of the Royal Statistical Society, which records that "Sir Henry was himself a member of the Council for several years, and on two occasions read a paper before the Society. He was also a generous donor to the library, where his works stand as a memorial of his many and fruitful activities." From the Grand Priory of the Order of the Hospital of St. John of Jerusalem in England, of which Sir Henry was a Knight of Grace. From the Board of Management of King Edward VII. Infirmary, Cardiff, in conveying whose resolution Mr. L. D. Rea, the General Superintendent, writes: "The community is very much the poorer by his death, and the hospitals especially lose in him a staunch and fearless friend and one who knew thoroughly what he was talking and writing about. It would require pages to write of his many activities, of his endless work and thought and hope for all things affecting hospitals, and one is apt to overlook, in considering the life of a great public man, his personal kindliness. Of that I can bear sincere testimony. I always found him ready to help, sympathetic and appreciative to a degree, and full of encouragement for every enterprise that merited his approval."

